# Urban–rural disparities in the association of nitrogen dioxide exposure with cardiovascular disease risk in China: effect size and economic burden

**DOI:** 10.1186/s12939-024-02117-3

**Published:** 2024-02-06

**Authors:** Yike Zhang, Mengxiao Hu, Bowen Xiang, Haiyang Yu, Qing Wang

**Affiliations:** 1 Department of Biostatistics, School of Public Health, Cheeloo College of Medicine, Shandong University, Jinan, Shandong 250012 China; 2 National Institute of Health Data Science of China, Shandong University, Jinan, China

**Keywords:** Nitrogen dioxide, Urban–rural disparities, Cardiovascular disease admissions, Economic burden, Social inequality, China

## Abstract

**Background:**

Together with rapid urbanization, ambient nitrogen dioxide (NO_2_) exposure has become a growing health threat. However, little is known about the urban–rural disparities in the health implications of short-term NO_2_ exposure. This study aimed to compare the association between short-term NO_2_ exposure and hospitalization for cardiovascular disease (CVD) among urban and rural residents in Shandong Province, China. Then, this study further explored the urban–rural disparities in the economic burden attributed to NO_2_ and the explanation for the disparities.

**Methods:**

Daily hospitalization data were obtained from an electronic medical records dataset covering a population of 5 million. In total, 303,217 hospital admissions for CVD were analyzed. A three-stage time-series analytic approach was used to estimate the county-level association and the attributed economic burden.

**Results:**

For every 10-μg/m^3^ increase in NO_2_ concentrations, this study observed a significant percentage increase in hospital admissions on the day of exposure of 1.42% (95% CI 0.92 to 1.92%) for CVD. The effect size was slightly higher in urban areas, while the urban–rural difference was not significant. However, a more pronounced displacement phenomenon was found in rural areas, and the economic burden attributed to NO_2_ was significantly higher in urban areas. At an annual average NO_2_ concentration of 10 μg/m^3^, total hospital days and expenses in urban areas were reduced by 81,801 (44,831 to 118,191) days and 60,121 (33,002 to 86,729) thousand CNY, respectively, almost twice as much as in rural areas. Due to disadvantages in socioeconomic status and medical resources, despite similar air pollution levels in the urban and rural areas of our sample sites, the rural population tended to spend less on hospitalization services.

**Conclusions:**

Short-term exposure to ambient NO_2_ could lead to considerable health impacts in either urban or rural areas of Shandong Province, China. Moreover, urban–rural differences in socioeconomic status and medical resources contributed to the urban–rural disparities in the economic burden attributed to NO_2_ exposure. The health implications of NO_2_ exposure are a social problem in addition to an environmental problem. Thus, this study suggests a coordinated intervention system that targets environmental and social inequality factors simultaneously.

**Supplementary Information:**

The online version contains supplementary material available at 10.1186/s12939-024-02117-3.

## Introduction

Under the circumstances of rapid urbanization, ambient nitrogen dioxide (NO_2_) exposure has become a growing health threat in China [1]. The combined pace of economic growth and urbanization has led to tremendous increases in energy consumption. Consequently, NO_2_ is a major ambient pollutant [2]. Ambient NO_2_ exposure is considered to lead to cardiovascular disease (CVD) via dozens of molecular alterations, including but not limited to systemic inflammation, oxidative stress, endothelial dysfunction, coagulation, lipid metabolism, and vascular smooth muscle cell proliferation [3]. A series of epidemiological studies have linked short-term NO_2_ exposure to various CVD risks, such as hypertension, coronary heart disease, stroke, arrhythmia, and dyslipidemia [4–8]. Notably, the urban–rural disparities in CVD incidence and morbidity are prominent in China. The most recent national survey revealed that the prevalence rates of CVD among rural and urban residents were 139.3 ‰ and 168.0 ‰, respectively, while the mortality rates were 323.29 and 277.92 per 100,000 rural and urban residents, respectively, and the mortality rate from CVD in rural regions had consistently surpassed and remained higher than that in urban areas since 2009. Thus, the extent to which short-term NO_2_ exposure may lead to disparities should be determined.

Due to the difficulty in obtaining air pollution data and resident health data in rural areas, most studies on the health implications of NO_2_ have been carried out in urban areas. Only a few studies have compared the association of NO_2_ with CVD risk between urban and rural areas, and the available results are mixed. A study identified that the health risks from short-term NO_2_ exposure increased with the urbanization process based on data from the Pearl River Delta region [9]. A comparative study of urban and rural areas in Guangxi Province, China, also suggested that the effects of NO_2_ on CVD hospitalizations were not significant in rural areas, whereas urban residents were significantly and negatively affected by NO_2_ exposure [10]. In contrast, Li et al. found that rural residents were more sensitive to short-term NO_2_ exposure than urban residents in terms of CVD mortality, but the differences were not significant [11]. Thus, additional research is warranted to better understand the urban–rural disparities in this association.

Social inequality between urban and rural residents may further contribute to urban–rural disparities in the association of nitrogen dioxide exposure with CVD risk in terms of effect size and economic burden. Although rural–urban differences in access to basic health care have narrowed in China, access to high-quality health care services persist due to dramatic urban–rural differences in socioeconomic status. Rural residents usually have limited access to high-quality health care services, which may cause delayed and even reduced treatment of CVD attributed to short-term NO_2_ exposure. Compared to their urban counterparts, the rural population ranks lower in socioeconomic status [12], which further constrains rural residents in affording high-quality health care and leads to urban–rural disparities in the economic burden attributed to NO_2_ exposure. As Mohai et al. reported, environmental health issues are not only an environmental problem but also connected to social inequality [13]. More studies are also needed to corroborate the extent to which to what extent social inequality in socioeconomic status and access to health care may influence the urban–rural disparities in the effects of NO_2_ exposure.

Therefore, the objective of this study was to compare the association between short-term NO_2_ exposure and hospitalization for CVD among urban and rural residents in Shandong Province, China. Then, this study further explored the urban–rural disparities in the economic burden attributed to NO_2_ and the potential explanations from the perspective of social inequalities. To our knowledge, this is the first study to assess the urban–rural disparities in the economic burden attributed to NO_2_ exposure. A better understanding of the urban–rural disparities in short-term NO_2_-related health risks is of great importance for effective and timely decision-making in designing spatially targeted health interventions and developing NO_2_-resilient health systems. Furthermore, this study provides evidence to effectively allocate urban and rural medical resources from the perspectives of environmental justice and social equality.

### Chinese urban–rural background

China has experienced rapid urbanization in the past five decades, which took approximately a hundred years for Western societies [14]. Along with the urbanization process, urban–rural health and social disparities are becoming dramatic [15]. These disparities are rooted in development patterns and policy systems [16]. In the process of urbanization beginning in the 1970s, a series of urban-biased policies were implemented in China to invest resources in urban areas, contributing to rapid development in these areas [17]. However, the urban control and exploitation of rural areas have led to the decline of rural areas and the formation of an urban–rural dual structure in terms of economic levels and access to welfare [15]. For example, the per capita disposable income of urban residents increased from CNY 343.4 in 1978 to CNY 6280.0 in 2000, while that of rural residents increased from CNY 133.6 to CNY 2253.4 during the same period [18]. Moreover, medical resources are mostly concentrated in urban areas. In 2000, the numbers of health technicians and beds per thousand people in rural areas were 2.41 and 1.50, respectively. The numbers of health technicians and beds in urban areas were 2.15 and 2.33 times those in rural areas, respectively [18]. However, air quality was much better in rural areas due to the slow industrialization process in this period [19].

Since 2000, urban–rural relations and the dependence of urban areas on rural areas have begun to change [14]. New types of urbanization and rural revitalization strategies have been put forward in succession, and urban and rural areas have exhibited a new trend of integrated development. Specifically, in rural areas, targeted poverty alleviation aims to lift all destitute households out of poverty and underdevelopment. Then, the Beautiful Countryside Plan can allow the improvement of infrastructure and social welfare in rural areas. Specifically, China has endeavored to achieve universal health coverage (UHC) and grant access for every citizen to equitable, accessible, and reliable health services and protection. To ensure that urban and rural residents enjoy equal access to basic medical care, China has established a medical security system covering 1.3 billion people, with a participation rate of over 95% [20]. Combined with the reform of public hospitals, a hierarchical medical system, the development of contracts with family doctors, and the basic drug system, China’s health care reform has achieved population coverage, service coverage, and cost coverage, which are the three dimensions of UHC realization [21].

However, due to industrialization and solid fuel emissions, air pollution is becoming a major problem in a growing number of rural areas. Moreover, the imbalance between rural and urban development continues to be prominent in many provinces [22]. Urban areas usually hold an advantage in socioeconomic status. According to the 2021 yearbook [23], the per capita disposable income of urban and rural residents was CNY 43833.8 and CNY 17131.5 in 2020, respectively. In addition to income inequality, urban–rural health inequality remains problematic. Despite similar access to basic medical care, high-quality health care resources are distributed mostly in urban areas [24, 25]. Compared with urban residents, the utilization of health services and annual health and hospitalization expenses are poorer for rural residents [12]. A higher mortality rate attributed to CVD has been documented in rural areas despite of a lower prevalence of CVD risk. Notably, spatial differences are dramatic in terms of urban–rural characteristics. For example, urban–rural differences in the NO_2_ annual average concentration exhibit a great gap in Chongqing and Guangxi but not in Shandong or Beijing [26]. A similar phenomenon was found regarding socioeconomic status and access to health care. Residents in rural areas of Guangdong and Hainan provinces are entitled to equal and even better health care than the average level. Thus, representative quantitative evidence is needed to reveal the associations between NO_2_ exposure and CVD risk in urban and rural areas.

## Methods

### Study population

This study derived data from the Cheeloo Lifespan Electronic Health Research Data Library (Cheeloo LEAD) using a three-stage cluster random sampling method. Cohorts from 39 counties were obtained, totaling 5 million individuals, sampled from 136 counties in Shandong Province, which has a total population of 101 million. The specific sampling process and the demographic characteristics of this population are provided in Supplementary Materials Fig. S1. A more detailed description of the study design and sampling procedure can be found at http://www.mhdata.sdu.edu.cn/cheeloolead.htm and in previously published studies [27–29]. Urban and rural areas are delineated based on the urban–rural categorization code established by the National Bureau of Statistics of China in 2015 (http://www.stats.gov.cn/sj/tjbz/qhdm/). This classification code comprises three numbers, where the initial digit being 1 indicates an urban area, and a first digit of 2 signifies a rural area. The 39 county-level units included 21 rural counties and 19 urban counties. The county names, county codes, and sample sizes of the sampled counties are shown in Table S1. Of them, Tengzhou city included an urban county and a rural county; thus, there were 39 county-level units.

### CVD hospital admission identification

Electronic medical records and medical insurance data were extracted for the sampled residents, and individual identification numbers and admission times were used as indexes to merge the information from the two data set. The essential hospital records of the study population included the names and codes of the discharge diagnosis, the length of hospitalization, and the names and expenses of prescriptions during hospitalization. A total of 1.7 million hospitalizations were extracted. Among them, 336,621 hospitalizations for CVD were screened according to International Statistical Classification of Diseases and Related Health Problems, 10th Revision (ICD-10) codes. After excluding a portion of missing data points, the final sample size was 303,127. Subsamples were further screened according to the ICD-10 (coronary heart disease: I20-I25; ischemic stroke: I63; hypertension: I10-I12). For a specific disease in a specific county on a given day, the county-level hospital admissions were obtained by summing the total number of hospital admissions on that day from the sampled population of that county. When calculating attributable hospital days and the burden of hospitalization costs, the total was derived by multiplying the average expenses and average length of stay for all admissions in a specific county during the study period by the attributable number of admissions for that county.

### Air pollution and meteorological data

Daily ambient NO_2_, particulate matter with an aerodynamic diameter of 2.5 μm or less (PM_2.5_), particulate matter with an aerodynamic diameter of 10 μm or less (PM_10_), sulfur dioxide (SO_2_), carbon monoxide (CO) and ozone (O_3_) data covering Shandong Province from 2015 to 2017 at a spatial resolution of 0.1° (≈10 km^2^) were collected from ChinaHighAirPollutants (CHAP, available at https://weijing-rs.github.io/product.html). These data are estimated by a space-time extremely randomized tree (STET) model. This model was developed to integrate satellite remote sensing products, atmospheric reanalysis, and ground-based measurements to complete model simulations. The pollutant estimations were reliable since they exhibited high R^2^ values of 0.80–0.91, with reference to surface observations obtained by adopting the independent 10-fold cross-validation approach. County-level data were extracted by averaging the grid values.

The daily mean temperature and relative humidity were based on the daily source data from a total of 131 meteorological monitoring stations in Shandong and adjacent provinces from the China Meteorological Data Sharing Service (http://data.cma.cn/). A thin-plate smooth spline function, with longitude and latitude as independent spline variables and elevation as a covariate considered in the function, was applied to interpolate the daily mean temperature and relative humidity grid at 0.01° * 0.01° resolution for the whole of Shandong Province from 2015 to 2017. County-level data were extracted by averaging the grid values.

## Statistical analysis

### Effect size and economic burden estimation

A three-stage time series design was used to estimate the association of short-term NO_2_ exposure with cardiovascular disease, coronary heart disease, stroke, and hypertension and the corresponding attributable hospital admissions, hospital days, and total hospital expenses.

In the first stage, a time series of a quasi-Poisson generalized linear regression model allowing for overdispersed admission counts was used to estimate county-specific associations [30].

1 $$Log\left(E\left({Y}_t\right)\right)=\alpha +\beta \left({NO}_2\right)+ Day\ of\ the\ week+ Holiday+ ns\left( calendar\ time, df=7\ per\ year\right)+ ns\left( temperature, df=6\right)+ ns\left( relative\ humidity, df=3\right)$$

In the model, several confounding covariates were incorporated, including daily mean temperature, relative humidity, calendar time, holiday, and day of the week, which were predefined according to previously published studies [31, 32]. In the equation, E(Y_t_) is the expected count of admissions in the analyzed county on day t, and β(NO_2_) is the log relative risk of hospital admissions associated with a 10-μg/m^3^ increase in NO_2_. Following Tian et al., the day of the week and holiday are the indicator variables to account for possible differences between weekdays and weekends and holidays and nonholidays. Previous studies have found variations in healthcare service utilization between weekdays and weekends [33], holidays and non-holidays [34]. In Chinese cultural context, people prefer not to receive health services on holidays (such as Spring Festival and Mid-Autumn Festival, which are usual days for family reunion). Moreover, in China, there could be overlap between the two variables, but few days on both holidays and weekends. ns (temperature) and ns (relative humidity) are natural cubic splines with 6 df for the 3-day moving average temperature and 3 df for the 3-day moving average relative humidity to adjust for potential lag and nonlinear effects effect of temperature and relative humidity; ns (calendar time) is a natural cubic spline function of time with seven degrees of freedom (df) per year to adjust for seasonality and time trends. Confounding effects of time-invariant or slowly varying risk factors at the individual level (e.g., sex, age, and comorbidities) could be naturally controlled for in the model [32].

This study modeled the association between NO_2_ and hospital admissions using a distributed lag model with a linear lag response function, inspecting the lag structure on a single lag day of 0 to 4 and moving average of the present and previous days (lag 0–4), respectively, to identify the optimal lag choices. In this model, lag 0 corresponded to the present day, lag 1 to the previous day, lag 2 to the day before lag 1, lag 3 to the day before lag 2, and lag 0–1 represented the two-day moving average of the present and previous day. The single-day and cumulative exposure effects were calculated based on exposures defined by these two different lag structures, and the estimated cumulative exposure effect was similar to the sum of the coefficients for single-day exposure effects in the distributed lag model [35].

In the second stage, random effects meta-analyses were applied to pool the county-specific associations to obtain urban, rural and overall estimates [36, 37]. The associations were calculated and expressed as the percentage change (95% CI) for each NO_2_ increase of 10 μg/m^3^.

2 $$RR={e}^{\beta }$$

3 $$Percentage\ change\ \left(\%\right)=\left( RR-1\right)\ast 100$$

A two-sample test was implemented to assess statistically significant differences in the estimates (E) between urban and rural areas based on the point estimate and standard error (SE) [38].

4 $$Z=\frac{E_{urban}-{E}_{rural}}{\sqrt{SE{\left({E}_{urban}\right)}^2+ SE{\left({E}_{rural}\right)}^2}}$$

In the third stage, the urban and rural effect estimates from the second stage were used to calculate the attributable number (AN) and attributable fraction (AF) [39] corresponding to the reduction in hospital admissions, length of hospital stays and total hospital expenses at the optimal lag choice period when NO_2_ concentrations reached the 2005 World Health Organization Global Air Quality Guidelines (WHO 2005 AQG) and WHO 2021 AQG, respectively.

5 $$A{N}_ic= N_ic\ast \frac{RR_ic-1}{RR_ic}, with\ {RR}_ic={e}^{\left(\beta \ast \frac{D_ic- AQG}{10}\right)}$$

where AN_i_c is the county-specific attributable number of hospital admissions; N_i_c is the annual total hospital admissions in year i for urban or rural county c; β is the coefficient derived from the second stage; Dic is the annual average concentration of NO_2_ in year i for county c; and AQG is the World Health Organization Air Quality Guidelines annual average concentration of NO_2_, which is 40 μg/m^3^ for the WHO 2005 AQG and 10 μg/m^3^ for the WHO 2021 AQG. Total AN is summed by AN_i_c. AF is calculated by dividing the AN by the sum of Nic.

In addition, the AFs and ANs of total hospital stays and expenses were estimated using the following formula [40, 41]:

6 $$ANec= AEc\ast \sum A{N}_ic, ANdc= ADc\ast \sum A{N}_ic$$

where ANec and ANdc are the county-specific attributable number of hospital expenses and hospital stays, respectively. AN_i_c is the AN of hospital admissions for county c during the study period. AEc and ADc are the average expenses and average length of stay for all admissions in county c during the study period. AF was calculated by dividing the AN by the sum of the total expenses.

### Potential reasons for urban–rural disparities

Finally, meta-regression models with county-level social characteristics (such as access to health care and GDP per capita) as independent variables were employed to check the role of social inequality in the urban–rural association. County-level association estimation could not adjust for individual-level risk factors for CVD, such as lifestyle factors and obesity. Instead, given the relationship of GDP with lifestyle and obesity, we attempted to explore the role of economic development in the association between NO_2_ exposure and CVD risk at lag day of 0. These methods expanded into multivariate meta-regression models with specific predictors to explain the potential heterogeneity, representing a refined parameterization within the linear mixed effects meta-analytic framework [42].

Moreover, health service utilization between rural and urban residents was compared to explore the potential explanation for urban–rural disparities in the economic burden attributed to NO_2_ exposure. In addition, this study followed up on the death outcome of the participants up to October 2020, and the survival curve of CVD between urban and rural residents was evaluated using Kaplan–Meier curves [43].

## Sensitivity analysis

Since older people are less likely to move between rural and urban areas, the association among older people was also evaluated to reduce the bias resulting from the dynamic movement of citizens between rural and urban areas. In addition, five other co-pollutants (PM_10_, PM_2.5_, SO_2_, CO, and O_3_) were added to fit the two-pollutant model. By doing so, this study could determine the independent effects of short-term exposure to NO_2_ on CVD admissions. Based on previous studies, the association between short-term exposure to NO_2_ and increased risk of hospitalization for CVD was assumed to be linear in the main model [7, 44]. To explore the potential nonlinear correlation, NO_2_ was adjusted for using natural cubic splines, two knots were set at concentrations of 20 μg/m^3^ and 40 μg/m^3^, and the meta-smoothing method was used to summarize the concentration–response relationship curves.

All statistical analyses were conducted in R software (version 4.2.0) using the tsModel and dlnm packages for fitting first-stage models, the mixmeta package for performing meta-analyses, and the survminer package for performing survival analysis.

## Results

### Descriptive statistics

Table 1 shows the summary statistics for total hospital admissions, lengths of stay, and total expenses for each estimated disease during the study period in both urban and rural areas. Among the 303,217 hospital admissions for CVD, coronary heart disease had the largest number of hospital admissions in total (85,168) and urban areas (50,487), while ischemic stroke had the largest number of hospital admissions in rural areas (37,276). The total hospital stays and hospital expenses reached 4,302,498 days and 2,750,867 CNY for CVD, respectively. In urban areas, the total number of hospital days (3,032,005 days) and hospital costs (175,769 thousand CNY) for CVD were higher than those in rural areas (127,493 days and 1,000,098 thousand CNY).

**Table 1:** Characteristics of hospital admission cases in the included Shandong counties

Disease	Total	Urban	Rural
**Hospital admissions (cases)**			
Cardiovascular disease	303,217	160,652	142,565
Coronary heart disease	85,168	50,487	34,681
Ischemic stroke	68,078	30,802	37,276
Hypertension	45,149	27,054	18,095
**Total hospital stays (days)**			
Cardiovascular disease	4,302,498	3,032,005	1,270,493
Coronary heart disease	895,039	618,506	276,533
Ischemic stroke	844,794	473,765	371,029
Hypertension	767,184	628,622	138,562
**Total expenses (thousand CNY)**			
Cardiovascular disease	2,750,867	1,750,769	1,000,098
Coronary heart disease	872,259	607,536	264,723
Ischemic stroke	553,455	320,728	232,727
Hypertension	235,917	177,565	5835

*Abbreviation*: *CNY* Chinese yuan

**Fig. 1:**
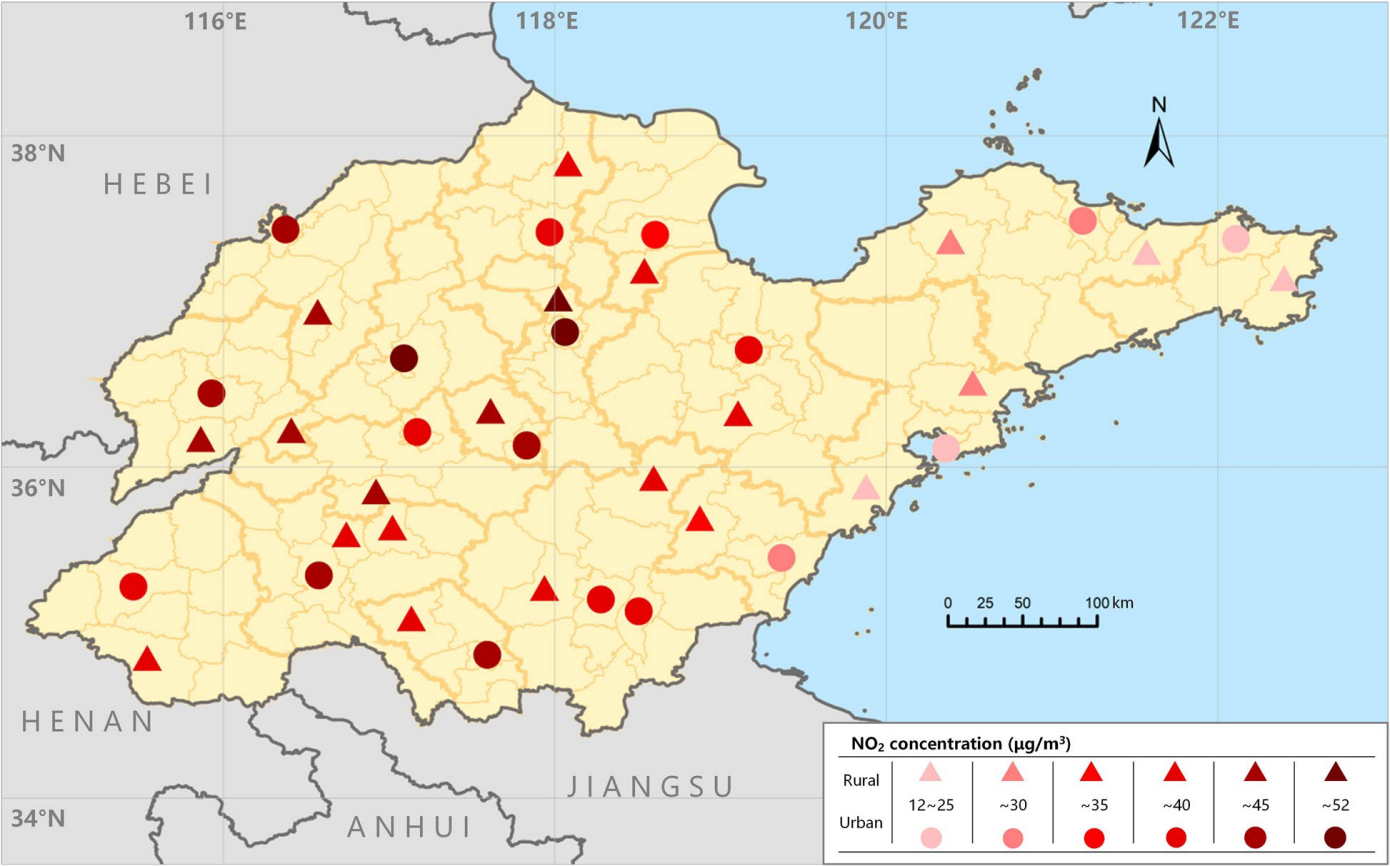
The distribution of 39 Shandong counties and their average nitrogen dioxide concentrations

Table 2 shows the statistical description of NO_2_ concentrations, temperature, and relative humidity in the study areas. The daily average NO_2_ concentration during the study period was 36.1 μg/m^3^, which was slightly higher in urban areas (37.2 μg/m^3^) than in rural areas (35.1 μg/m^3^). The daily average concentration of NO_2_ ranged from 14.74 to 93.78 μg/m^3^ in urban areas. This value ranged from 14.49 to 87.00 μg/m^3^ in rural areas. Figure 1 displays the geographical distribution of the sampled urban and rural areas within Shandong Province, along with the distribution of average NO_2_ concentrations. This shows that the distribution of the sampling points was quite dispersed. In addition, ambient NO_2_ pollution intensified from east to west, a pattern that is consistent with the distribution characteristics observed across China. Among our sample sites, daily NO_2_ concentrations were similar in urban and adjacent rural areas of coastal cities such as Qingdao, Yantai, and Weihai, whereas compared to adjacent rural areas, the air pollution levels of urban areas were obviously higher in some inland cities such as Jinan, Zaozhuang and Linyi. Furthermore, county-level hospital admissions, along with air pollution levels, socioeconomic factors, and health care resource indicators, are shown in Table S2. Overall, GDP per capita and number of beds per thousand people were higher in urban areas.

**Table 2:** Description of daily air pollutant concentrations, temperature and relative humidity in the included Shandong counties

	Mean (SD)	Median (P25, P75)
**NO**_**2**_ **(μg/m**^**3**^**)**		
Total	36.12 (15.97)	33.39 (24.61, 45.00)
Urban	37.20 (16.36)	34.31 (25.25, 46.21)
Rural	35.14 (15.40)	32.57 (24.12, 43.75)
**PM**_**2.5**_ **(μg/m**^**3**^**)**		
Total	63.31 (39.83)	53.47 (36.77, 77.93)
Urban	64.58 (41.24)	54.31 (37.26, 79.52)
Rural	62.47 (38.44)	53.03 (36.71, 76.87)
**PM**_**10**_ **(μg/m**^**3**^**)**		
Total	112.27 (57.95)	101.72 (72.05, 137.38)
Urban	115.23 (60.42)	103.84 (73.51, 141.51)
Rural	109.99 (55.33)	100.30 (71.15, 134.49)
**CO (μg/m**^**3**^**)**		
Total	1.19 (0.59)	1.09 (0.81, 1.43)
Urban	1.21 (0.62)	1.10 (0.81, 1.45)
Rural	1.17 (0.56)	1.08 (0.81, 1.40)
**O**_**3**_ **(μg/m**^3^**)**		
Total	82.52 (99.46)	91.35 (54.91, 132.29)
Urban	81.48 (105.02)	90.50 (53.54, 131.89)
Rural	84.72 (92.30)	92.84 (56.85, 133.19)
**SO**_**2**_ **(μg/m**^**3**^**)**		
Total	33.16 (22.34)	27.62 (16.92, 43.42)
Urban	33.63 (23.33)	27.58 (16.92, 43.66)
Rural	33.09 (21.32)	28.08 (17.35, 43.77)
**Temperature (°C)**		
Total	14.83 (10.01)	16.66 (5.6, 23.53)
Urban	14.87 (10.04)	16.74 (5.61, 23.60)
Rural	14.83 (9.98)	16.62 (5.65, 23.50)
**Relative humidity (%)**		
Total	65.83 (16.24)	66.84 (53.41, 79.02)
Urban	65.71 (16.29)	66.72 (53.24, 79.11)
Rural	66.12 (16.14)	67.14 (53.87, 79.12)

*Abbreviations*: *NO*_*2*_ nitrogen dioxide, *PM*_*2.5*_ particulate matter with an aerodynamic diameter of 2.5 μm or less, *PM*_*10*_ particulate matter with an aerodynamic diameter of 10 μm or less, *CO* carbon monoxide, *O*_*3*_ ozone, *SO*_*2*_ sulfur dioxide, *SD* standard deviation, *P25* 25th percentile, *P75* 75th percentile

### Associations between NO_2_ exposure and hospital admissions for CVD

**Fig. 2:**
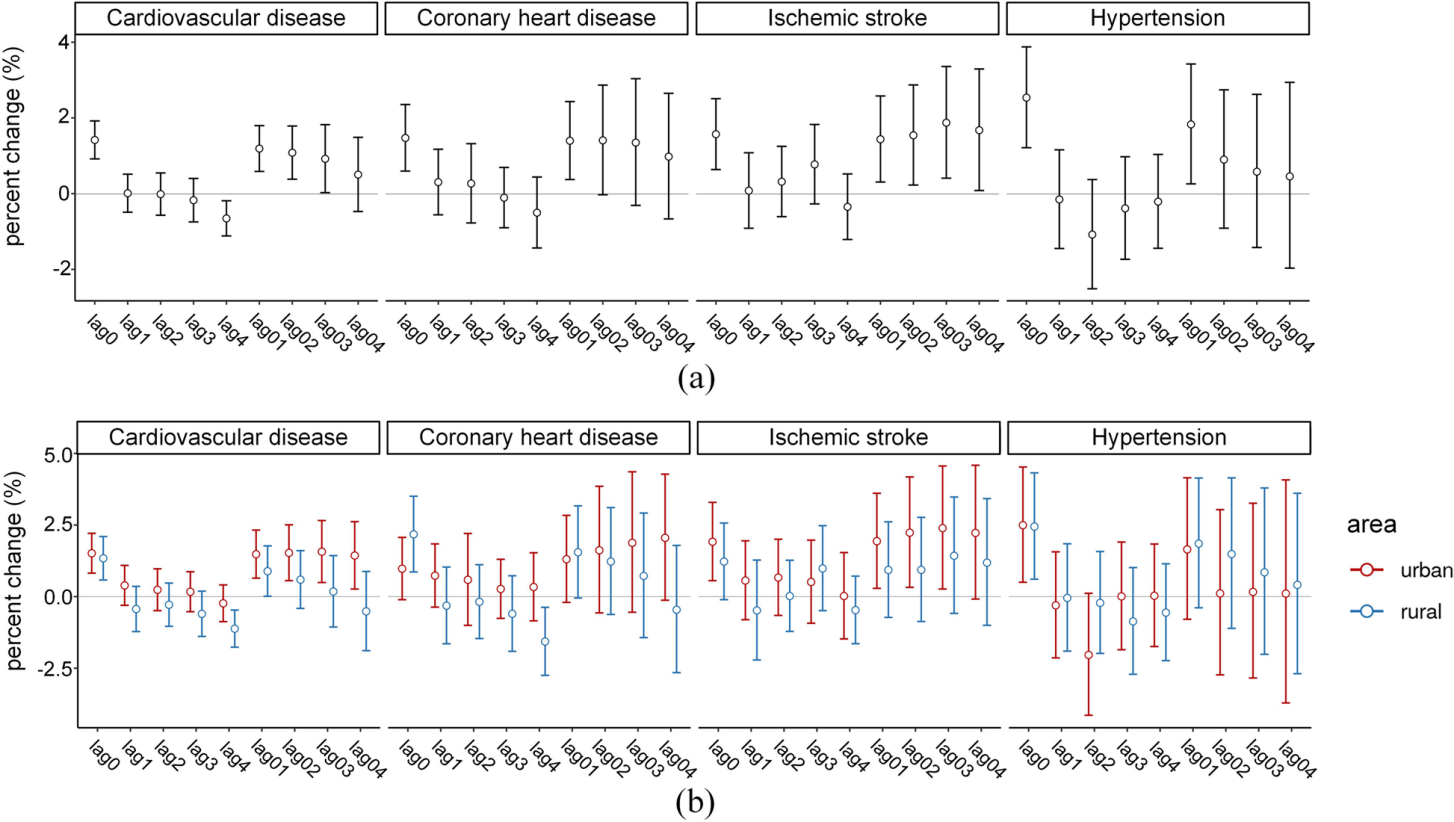
Percentage increase in cardiovascular disease, coronary heart disease, ischemic stroke, and hypertension hospital admissions per 10-μg/m^3^ increase in short-term ambient nitrogen dioxide exposure for overall (**a**) rural and urban counties (**b**). Note: The results were controlled for risk factors, including daily mean temperature, relative humidity, calendar time, public holidays, and day of the week. A distributed lag model was applied to estimate the county-specific associations, while random effects meta-analyses were used to pool the county-specific associations. Lag 0 corresponded to the present day, lag 1 to the previous day, lag 2 to the day before lag 1, lag 3 to the day before lag 2, and lag 0–1 represented the two-day moving average of the present and previous day

Figure 2  shows the total, urban, and rural estimates of the associations between NO_2_ and hospital admissions for CVD as well as coronary heart disease, ischemic disease, and hypertension on different lag days (including single-day lags and cumulative lags from 0 to 4 days). For the total effect estimates, a similar lagged pattern, namely, a significant and almost highest estimate at lag 0, was exhibited for total CVD and for the other three cause-specific diseases. For a 10-μg/m^3^ increase in NO_2_ concentrations, this study observed a significant percentage increase in hospital admissions on the day of exposure of 1.42% (95% confidence interval 0.92 to 1.92%) for CVD, 1.47% (0.59 to 2.35%) for coronary heart disease, 1.57% (0.64 to 2.51%) for ischemic stroke, and 2.54% (1.21 to 3.88%) for hypertension. However, as the lag day length increased, the single-day effect of cardiovascular disease began to show a protective effect, and the cumulative effect gradually decreased and was no longer significant. This is referred to as the ‘displacement’ phenomenon by Schwartz [45].

### Urban–rural disparities in the association between NO_2_ exposure and hospital admissions for CVD

The comparison of the urban and rural estimates is shown in Fig. 2(b). Separate estimates in urban and rural areas also suggested a significant effect on hospital admissions for CVD at lag 0, with 1.51% (0.82 to 2.21%) and 1.34% (0.58 to 2.10%), respectively. The estimated effects were nonsignificantly different for urban and rural counties (Table 3) at lag 0 (*P* value = 0.738). Similarly, the ‘displacement’ phenomenon was observed in the urban and rural estimates. Nevertheless, a more pronounced displacement phenomenon was found in rural areas, with a statistically significant difference (urban vs. rural: 1.43% (0.26 to 2.62%) vs. − 0.52% (− 1.89 to 0.88%), *P* value = 0.035). In addition, the results for the three cause-specific CVDs differed between urban and rural areas. For a 10-μg/m^3^ increase in NO_2_ concentrations, there was a significant percentage increase in hospital admissions on the day of exposure of 1.92% (0.56 to 3.29%) for ischemic stroke and 2.49% (0.50 to 4.53%) for hypertension among urban residents, while there was a significant increase of 2.18% (0.86 to 3.51%) for coronary heart disease and 2.45% (0.61 to 4.32%) for hypertension among rural residents.

**Table 3:** Percentage changes in hospital admissions associated with a 10-μg/m^3^ increase in NO_2_ on lag 0 days and lag 04 days

	Percentage change in % (95% CI)	
	Lag 0	Lag 04
**Cardiovascular disease**		
Total	1.42 (0.92, 1.92)	0.50 (−0.47, 1.49)
Urban	1.51 (0.82, 2.21)	1.43 (0.26, 2.62)
Rural	1.34 (0.58, 2.10)	−0.52 (−1.89, 0.88)
p*	0.738	0.035
**Coronary heart disease**		
Total	1.47 (0.59, 2.35)	0.98 (−0.67, 2.65)
Urban	0.97 (−0.11, 2.07)	2.05 (− 0.13, 4.28)
Rural	2.18 (0.86, 3.51)	−0.46 (−2.66, 1.79)
p*	0.169	0.116
**Ischemic stroke**		
Total	1.57 (0.64, 2.51)	1.68 (0.08, 3.29)
Urban	1.92 (0.56, 3.29)	2.22 (−0.08, 4.59)
Rural	1.22 (−0.11, 2.57)	1.19 (−1.00, 3.42)
p*	0.477	0.527
**Hypertension**		
Total	2.54 (1.21, 3.88)	0.46 (−1.97, 2.94)
Urban	2.49 (0.50, 4.53)	0.11 (−3.71, 4.08)
Rural	2.45 (0.61, 4.32)	0.41 (−2.69, 3.61)
p*	0.973	0.907

* The *p* values were for difference tests in the associations between urban and rural areas

Lag 0 corresponded to the present day, and lag 0–4 represented the four-day moving average of the present and previous day

The pooled concentration–response curve (Fig. S2) for the association between NO_2_ and hospital admissions showed positive and nearly linear concentration–response curves, with no discernible thresholds. In the sensitivity analysis, models with two pollutants were used. The associations of NO_2_ with total and cause-specific CVDs were still robust after adjustment for co-pollutants (Table 4). However, the effect size of NO_2_ increased slightly after adjustment for PM_2.5_, PM_10_, SO_2_, O_3_ and CO. Fig. S3 shows the comparison between the results of subsample analysis in the aging population (aged above 60, *n* = 214,792) and of all study subjects. Since older people are less likely to move between rural and urban areas, the results for the total population were very similar to those in the aging population, suggesting that the dynamic movement of citizens between rural and urban areas did not bias our estimations.

**Table 4:** Percentage changes in cardiovascular disease, coronary heart disease, ischemic stroke, and hypertension hospital admissions associated with a 10-μg/m^3^ increase in NO_2_ on lag 0 days, with and without adjustment for co-pollutants

Model	Disease	Percentage change in % (95% CI)	Attributable fraction in % (95% CI)
**Adjusting for PM2.5**			
	Cardiovascular disease	2.20 (1.44, 2.96)	7.16 (4.78, 9.48)
	Coronary heart disease	2.45 (1.21, 3.70)	8.10 (4.13, 11.89)
	Ischemic stroke	1.79 (0.29, 3.31)	6.21 (1.04,11.10)
	Hypertension	3.68 (1.50, 5.9)	10.82 (4.64, 16.57)
**Adjusting for PM10**			
	Cardiovascular disease	1.92 (1.27, 2.57)	6.29 (4.23, 8.29)
	Coronary heart disease	2.25 (1.14, 3.36)	7.46 (3.90, 10.88)
	Ischemic stroke	1.96 (0.78, 3.16)	6.80 (2.78, 10.65)
	Hypertension	2.95 (1.02, 4.92)	8.83 (3.19, 14.11)
**Adjusting for CO**			
	Cardiovascular disease	2.20 (1.22, 3.19)	7.18 (4.08, 10.17)
	Coronary heart disease	2.66 (1.36, 3.98)	8.77 (4.63, 12.72)
	Ischemic stroke	1.11 (−0.61, 2.86)	3.91 (−2.25, 9.69)
	Hypertension	4.90 (2.46, 7.40)	14.07 (7.44, 20.18)
**Adjusting for O3**			
	Cardiovascular disease	1.37 (0.86, 1.87)	4.54 (2.90, 6.14)
	Coronary heart disease	1.41 (0.53, 2.29)	4.78 (1.85, 7.62)
	Ischemic stroke	1.40 (0.46, 2.34)	4.90 (1.65, 8.03)
	Hypertension	2.42 (1.05, 3.81)	7.30 (3.26, 11.17)
**Adjusting for SO**_**2**_			
	Cardiovascular disease	1.81 (1.06, 2.57)	5.95 (3.53, 8.31)
	Coronary heart disease	1.89 (0.63, 3.16)	6.34 (2.19, 10.30)
	Ischemic stroke	2.16 (0.77, 3.58)	7.45 (2.74, 11.93)
	Hypertension	2.45 (0.28, 4.66)	7.40 (0.89, 13.44)

*Abbreviations*: *PM*_*2.5*_ particulate matter with an aerodynamic diameter of 2.5 μm or less, *PM*_*10*_ particulate matter with an aerodynamic diameter of 10 μm or less, *CO* carbon monoxide, *O*_*3*_ ozone, *SO*_*2*_ sulfur dioxide

### Economic burden attributed to NO_2_ exposure in terms of CVD

Table 5 shows the AFs and ANs of hospital admissions, total hospital days, and total expenses that could be reduced if annual NO_2_ concentrations reached the WHO 2021 AQG. By doing so, this study could reflect the disease burden and economic burden of CVD caused by NO_2_. At an annual average NO_2_ concentration of 10 μg/m^3^ (WHO 2021 AQG), the reduced AN of CVD hospital admissions would be 5447 (2990 to 7859) in urban areas, with an AF of 3.39% (1.86 to 4.89%). The results in rural areas were very similar to those in urban areas, with AN and AF values of 4765 (2099 to 7376) and 3.34 (1.47 to 5.17), respectively. However, there were apparent urban–rural differences in length of stay and hospital expenses. Total hospital days and expenses in urban areas would be reduced by 81,801 (44,831 to 118,191) days and 60,121 (33,002 to 86,729) thousand CNY, respectively, almost twice as much as in rural areas, with 42,131 (18,562 to 65,212) days and 32,875 (14,483 to 50,886) thousand CNY, respectively. Hypertension showed similar results: the burden of disease in urban areas was similar to that in rural areas, but the economic burden was almost twice as high. The previous WHO 2005 AQG standard set an annual average value of 40 μg/m^3^ for NO_2_, and this study also calculated the ANs and AFs of hospital admissions, hospital days, and hospital expenses for this guideline in both urban and rural areas (Table S4). Since many of our study areas already met this level, this guideline value would have resulted in a smaller reduction in both the disease and economic burden from NO_2_. The AN and AF were 327 (179 to 475) and 0.46% (0.25 to 0.66%) for hospital admissions for CVD in urban areas and 185 (81 to 289) and 0.26% (0.11 to 0.40%) in rural areas, respectively.

**Table 5:** Attributable numbers and fractions of hospital admissions, total hospital stays and total expenses (thousand CNY) that can be reduced when the annual NO_2_ concentration reaches the WHO 2021 AQG

	Attributable number (95% CI)		Attributable fraction in % (95% CI)	
	Urban	Rural	Urban	Rural
**Cardiovascular disease**				
Hospital admissions (case)	5447 (2990, 7859)	4765 (2099, 7376)	3.39 (1.86, 4.89)	3.34 (1.47, 5.17)
Total hospital stays (days)	81,801 (44,831, 118,191)	42,131 (18,562, 65,212)	2.70 (1.48, 3.90)	3.32 (1.46, 5.13)
Total expenses (thousand CNY)	60,121 (33,002, 86,729)	32,875 (14,483, 50,886)	3.43 (1.88, 4.95)	3.29 (1.45, 5.09)
**Coronary heart disease**				
Hospital admissions (case)	1171 (−133, 2434)	1893 (769, 2977)	2.32 (−0.26, 4.82)	5.46 (2.22, 8.58)
Total hospital stays (days)	12,728 (−1444, 26,501)	15,019 (6098, 23,616)	2.06 (−0.23, 4.28)	5.43 (2.21, 8.54)
Total expenses (thousand CNY)	13,950 (−1584, 29,011)	14,366 (5833, 22,592)	2.30 (−0.26, 4.78)	5.43 (2.20, 8.53)
**Ischemic stroke**				
Hospital admissions (case)	1449 (433, 2426)	1198 (−108, 2455)	4.70 (1.41, 7.88)	3.21 (−0.29, 6.59)
Total hospital stays (days)	19,803 (5915, 33,202)	11,809 (−1064, 24,207)	4.18 (1.25, 7.01)	3.18 (−0.29, 6.52)
Total expenses (thousand CNY)	14,575 (4357, 24,413)	7302 (− 658, 14,969)	4.54 (1.36, 7.61)	3.14 (−0.28, 6.43)
**Hypertension**				
Hospital admissions (case)	1272 (265, 2232)	1066 (274, 1819)	4.70 (0.98, 8.25)	5.89 (1.51, 10.05)
Total hospital stays (days)	22,999 (4760, 40,576)	8104 (2083, 13,831)	3.66 (0.76, 6.45)	5.85 (1.5, 9.98)
Total expenses (thousand CNY)	8435 (1756, 14,795)	3372 (867, 5756)	4.75 (0.99, 8.33)	5.78 (1.49, 9.86)

*Abbreviations*: *CNY* Chinese yuan, *NO*_*2*_ nitrogen dioxide, *WHO 2021 AQG the 2021* World Health Organization Global Air Quality Guidelines, *CI* confidence interval

### Exploration of the reasons behind urban–rural disparities

Table S3 shows the results of the meta-regression model adjusting for county-level access to health care and economic development. The estimated heterogeneity (I^2^) in the overall exposure–response associations for CVD hospital admissions at lag day of 0 between counties was 13.8%. Adding the GDP per capita and hospital beds per thousand people indicators to the model increased the model’s heterogeneity to 16.0% and decreased it to 12.8% respectively, with the *P* values of the coefficient estimates not being significant. The results suggest that the association between NO_2_ exposure and CVD risks at lag day of 0, when the effects are most pronounced, was not shaped by access to health care or economic development.

**Fig. 3:**
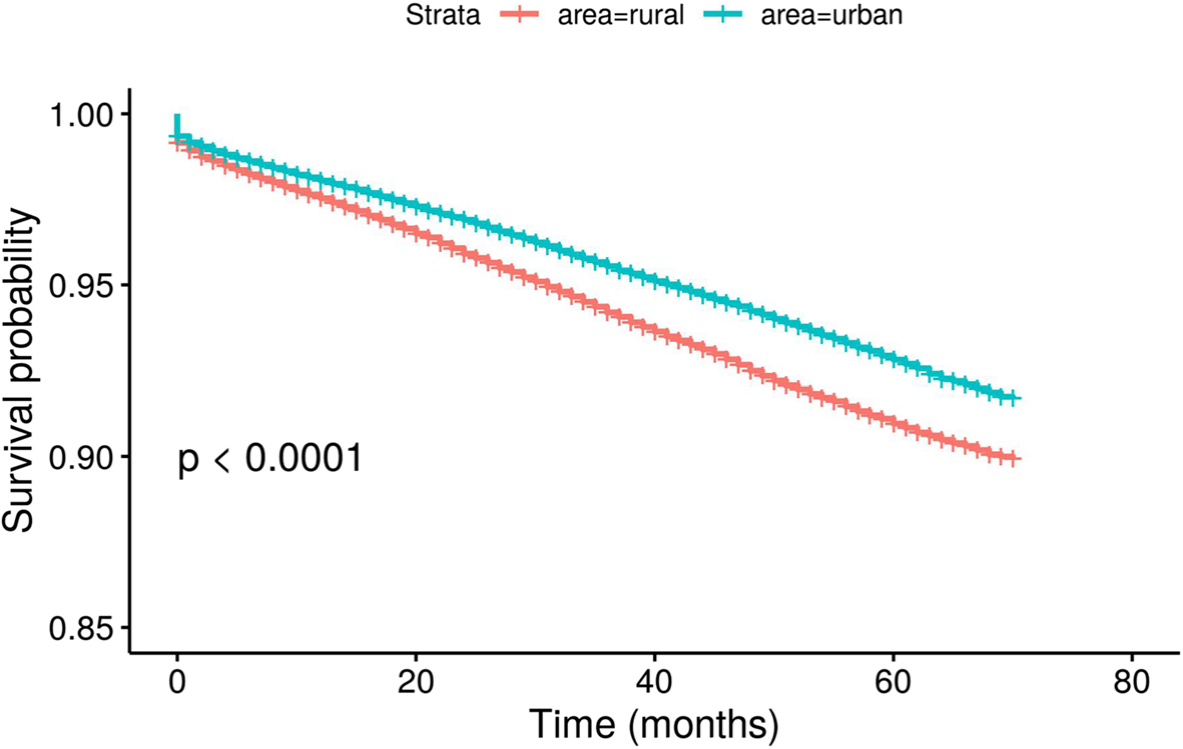
Kaplan–Meier survival curve of CVD between urban and rural residents

However, urban–rural differences in access to health care contributed to urban–rural disparities in the economic burden attributed to NO_2_ exposure. Table 6 displays the comparison of health service utilization between rural and urban residents. Rural residents tended to receive health care services in primary and secondary institutions and spent less per admission. In this study, 34.05 and 33.88% of rural patients received treatment in primary and secondary medical institutions, respectively, compared with 19.30 and 25.98% of urban patients. On average, total expenses per admission were lower among rural residents (urban vs. rural: 6826 CNY vs. 3854 CNY); when stratified by facility level, the differences became significantly smaller. Usually, tertiary institutions are more likely to provide high-quality health services, with a higher expense [46]. Thus, the urban–rural disparities in the economic burden attributed to NO_2_ exposure might be due to differences in access to high-quality health services between urban and rural residents. This study further tracked the mortality outcomes to support that urban residents had a high access to high-quality health services. The mortality outcomes of the study subjects was tracked until October 2020, and the Kaplan–Meier survival curve showed that there was a significant difference in the CVD survival time between urban and rural residents (Fig. 3). Urban residents showed a higher CVD survival probability, suggesting urban residents had a high access to high-quality health services. Since urban-rural difference in access to health services contributed to urban–rural disparities in the economic burden attributed to NO_2_ exposure, urban–rural disparities in the effects of NO_2_ exposure are a social problem in addition to environmental justice.

**Table 6:** Comparison of health service utilization between rural and urban residents

	Urban	Rural
**Admissions (proportion%)**		
Total	160,652	142,565
Primary medical institutions	31,011 (19.30%)	48,539 (34.05%)
Secondary medical institutions	41,736 (25.98%)	48,308 (33.88%)
Tertiary medical institutions	78,544 (48.89%)	32,861 (23.05%)
**Hospital expenses (CNY)**		
Total medical institutions	6826.28 (3796.62, 11,124.45)	3854.51 (2205.00, 6782.75)
Primary medical institutions	2652.20 (1547.23, 4395.18)	2290.60 (1559.78, 3232.86)
Secondary medical institutions	6430.90 (4296.72, 9239.11)	5044.75 (3392.34, 7674.43)
Tertiary medical institutions	9459.36 (6092.83, 14,781.79)	6823.405 (4286.33, 11,738.16)
**GDP per capita (CNY)**		
2015	77,812	71,405
2016	81,217	77,299
2017	88,627	81,689

*Abbreviation*: *CNY* Chinese yuan

## Discussion

### The association between short-term NO_2_ exposure and hospitalization for CVD

Based on data on 303,217 hospital admissions for CVD, this study estimated the urban–rural disparities in the association between short-term NO_2_ exposure and hospitalization for CVD in Shandong Province, China. As expected, this study found that NO_2_ was positively and significantly associated with hospitalization for CVD. For every 10-μg/m3 increase in NO_2_ concentrations, there were significant percentage increases in hospital admissions on the day of exposure of 1.42% (95% confidence interval 0.92 to 1.92%) for CVD, 1.47% (0.59 to 2.35%) for coronary heart disease, 1.57% (0.64 to 2.51%) for ischemic stroke, and 2.54% (1.21 to 3.88%) for hypertension. The estimations were on par with those of studies conducted in China [4, 6, 47–49] but greater than those in a global meta-analysis [8]. The meta-analysis revealed that a 10-μg/m3 increase in 24-h NO_2_ exposure was associated with a 0.66% (0.32 to 1.01%) increase in CVD hospital admissions. Nevertheless, the meta-analysis included 204 studies up to 2011, of which there were only 2 studies from East Asia.

This study further conducted estimations on the economic burden attributed to NO_2_ exposure. If the concentration of NO_2_ could be reduced to the WHO 2021 AQG standard (10 μg/m3), the AN of CVD hospital admissions that could be reduced would be 10,162 (6638 to 13,637), with attributed hospital expenses of 91,269 (59,627 to 122,479) thousand CNY. Compared to the WHO AQG for 2021, the reduced ANs and economic burden according to the WHO AQG for 2005 were obviously smaller. This could be because more than half of our sample sites met the WHO 2005 AQG criteria. In 2017, there were only three counties that did not meet the criteria. However, our study continued to observe the health hazards resulting from NO_2_ exposure, which justifies the importance of the WHO 2021 AQG.

### Urban–rural disparities in the association between short-term NO_2_ exposure and CVD hospital admissions

Separate estimations for urban and rural areas also suggested a significant effect of short-term NO_2_ exposure on CVD hospital admissions. A 10-μg/m^3^ increase in 24-h NO_2_ exposure was associated with 1.51% (0.82 to 2.21%) and 1.34% (0.58 to 2.10%) increases in CVD hospital admissions in rural and urban areas, respectively. However, the estimated effects were not significantly different for urban and rural counties. The results were consistent with those of Li et al. [13], who assessed urban–rural disparities in Beijing. In addition, the results were in agreement with those of Lin et al. [50] and Liu et al. [51], who compared the associations of short-term PM_2.5_/ozone with mortality (total, CVD, CED, and RESP) between urban cities and rural areas in Jiangsu and all of China. Conversely, no associations were found between short-term NO_2_ exposure and CVD mortality in urban or rural areas of Italy [52]. Moreover, a positive association between NO_2_ exposure and cardiovascular hospitalizations at lag 0 and lag 1 was found in urban areas of Guangxi Province [10], whereas the effect of NO_2_ was not significant in rural areas. The study populations had different urban–rural characteristics in terms of NO_2_ exposure across studies, which may have led to the differences in the results. For example, the rural areas of Italy and Guangxi were both characterized by low-level NO_2_ exposure, whereas the NO_2_ concentrations in the rural and urban areas of Shandong, Beijing, and Jiangsu were very similar.

However, a more pronounced displacement phenomenon was found in rural areas. The displacement phenomenon, referring to an increased risk ratio at short lags followed by an apparently protective effect at longer lags. This suggests that highly vulnerable people who are admitted to the hospital due to CVD may have simply had their problem brought forward by a few days as a result of NO_2_ exposure. Rural residents tended to have poorer overall health condition with lower health awareness compared to their urban counterparts, thus the displacement phenomenon might be greater among rural residents. This phenomenon has been observed for air pollution and temperature-related deaths [35, 45].

### Urban–rural differences in the economic burden attributed to NO_2_ exposure

Despite similar effect sizes, hospital days and expenses resulting from NO_2_ exposure in urban areas were almost twice as high as those in rural areas. This fact may demonstrate that social inequality and environmental justice may be interrelated. First high-quality medical resources are mainly distributed in urban areas. Rural residents have limited access to high-quality health care compared to urban residents and tend to receive health care in primary and secondary medical institutions. Correspondingly, expenses are generally lower for primary and secondary medical institutions than for tertiary institutions. In addition, a low socioeconomic status constrains rural residents’ health investments. To avoid catastrophic medical expenditures, rural residents may reduce hospital days and expenses [53]. Of course, our study could not clarify the potential explanation. Thus, additional research is warranted to better understand precisely how these differences may contribute to health disparities between urban and rural areas. However, based on our results, urban–rural disparities in the effects of NO_2_ exposure are a social problem in addition to environmental justice.

### Contributions and limitations

Based on the effects of short-term NO_2_ exposure on CVD hospitalization, this study also included the urban–rural disparities in the economic burden attributed to NO_2_ exposure. Many studies have evaluated the range of economic burdens associated with health problems caused by air pollution and made specific estimates [54–59]. Nevertheless, to our knowledge, no studies have compared these estimates between urban and rural areas. In addition, this study linked urban–rural disparities in environmental health with social inequality, which may enhance our understanding of the urban–rural disparities and the differences in previous studies. Our findings can help to provide justification for a coordinated intervention system that targets environmental factors and socioeconomic inequality simultaneously.

However, our study has several limitations. First, exposure misclassification could have occurred in our time series study design. Specifically, (i) county-level daily ambient air pollution could not exactly reflect personal exposure, and (ii) due to limited available data, we could not identify the divergence resulting from indoor-outdoor exposure. Since rural residents are more likely to be exposed to indoor NO_2_, the effect size of ambient NO_2_ exposure may be overestimated in rural areas, and urban-rural disparities in the effects of ambient NO_2_ exposure may be underestimated. However, disparities in the economic burden may remain. Second, the study area covered only Shandong Province, China. However, from east to west in Shandong Province, the environmental and sociodemographic characteristics are similar to those in China as a whole. Data spanning a wide range of economies and environments provide a good sample for analyzing the association. Third, our study sample was selected separately from urban community populations in municipal districts and rural villagers in rural counties to better compare typical rural–urban differences; however, this may have led to an overestimation of urban–rural disparities. Finally, our study was a time series observational study and could not control for sufficient variables; thus, this study could not explain the pathways of this phenomenon. However, we stratified our sample according to age and employed meta-regression models with the inclusion of access to health services and GDP to capture more factors influencing CVD risks. Due to the above limitations, our results should be interpreted with caution.

### Policy implications

Our investigation can provide guidance for the creation and implementation of prevention and intervention programs to mitigate the negative effects of short-term NO_2_ exposure. We need to attach importance to the early warning of NO_2_ pollution in the cardiovascular system. NO_2_ pollution in rural areas, which has often been neglected in the past, should receive special attention. In addition, a coordinated environmental health policy linking environmental and social factors with health is highly recommended to reduce the interaction between environmental inequities and social inequality. Mitigation and adaptation such as enhancing equity in access to health services and socioeconomic status, should be implemented in an integrated way. Finally, considering that dozens of counties meet the 2005 AQG standard but continue to be affected by NO_2_ exposure in terms of health, the WHO 2021 AQG should be widely adopted, and the guidelines should be dynamically adjusted.

## Conclusion

Based on data on 303,217 hospital admissions for CVD in Shandong Province, China, this study applied a distributed lag model and random effects meta-analyses to estimate the short-term association between NO_2_ exposure and hospitalization for CVD in both rural and urban areas. Afterward, urban–rural differences in the AN and AF attributed to NO_2_ were compared to reveal urban–rural disparities in the economic burden of CVD attributed to NO_2_ exposure. This study found a positive and significant association. Although the effect size was slightly higher in urban areas, the urban–rural difference was not significant. Nevertheless, a more pronounced displacement phenomenon was found in rural areas, and hospitalization expenses were significantly higher in urban areas. Differences in access to high-quality health care and socioeconomic status may partly explain the urban–rural disparities in the economic burden. Urban–rural disparities in the health implications of short-term NO_2_ exposure are a social problem in addition to an environmental problem. Thus, we may need to pay special attention to these rural areas in terms of the health implications of NO_2_ exposure, and a coordinated environmental health policy linking environmental and social factors with health should be implemented. However, our results should be applied with caution because of potential measurement error of exposure assessment, and lack of risk factors.

### Supplementary Information

**Additional file 1: Table S1.** County names, county codes and sample sizes of the included Shandong counties. **Table S2.** Hospital admissions, socioeconomic and health care accessibility indicators, and ambient NO_2_ concentrations of each area during the study period. **Table S3.** Meta-regression results for per capita GDP and hospital beds per thousand people of 39 counties in two-stage model analysis. **Table S4.** Attributable numbers and fractions of hospital admissions, total hospital stays and total expenses (thousand CNY) that can be reduced when the annual NO_2_ concentration reaches the WHO 2005 AQG. **Fig. S1.** Three-stage cluster sampling process, demographic characteristics of the sample population, and sample selection flowchart. **Fig. S2.** Concentration–response curve between NO_2_ concentrations (lag 0) and cardiovascular disease (a), coronary heart disease (b), ischemic stroke (c), and hypertension (d) hospital admissions. The vertical scale can be interpreted as the relative change in the mean effect of NO_2_ on mortality; the fraction of the curve below zero denotes a smaller estimate than the mean effect. **Fig. S3.** Comparison between the results of (a) subsample analysis in the aging population (aged above 60) and among (b) all study subjects.

## Data Availability

The datasets generated and/or analyzed during the current study are not publicly available to preserve individuals’ privacy.

## Abbreviations

AF: Attributable fraction
AN: Attributable number
CHAP: China high air pollutants dataset
95% CI: 95% Confidence interval
CNY: Chinese yuan
CO: Carbon monoxide
CVD: Cardiovascular disease
ICD-10: International statistical classification of diseases and related health problems 10th revision
NO_2_: Nitrogen dioxide
O_3_: Ozone
PM_2.5_: Particulate matter with an aerodynamic diameter of 2.5 μm or less
PM_10_: Particulate matter with an aerodynamic diameter of 10 μm or less
PPS: Probability proportional to size
STET: Space-time extremely randomized tree
SO_2_: Sulfur dioxide
UHC: Universal health coverage
WHO 2005 AQG: 2005 World health organization global air quality guidelines
WHO 2021 AQG: 2021 World health organization global air quality guidelines
