# Supplementary material for: Urban–rural disparities in the association of nitrogen dioxide exposure with cardiovascular disease risk in China: effect size and economic burden
Source: Int J Equity Health. 2024 Feb 6;23:22. doi: 10.1186/s12939-024-02117-3 (PMC10845777; doi:10.1186/s12939-024-02117-3)
Supplement: Supplementary file 1 — Additional file 1: Table S1. County names, county codes and sample sizes of the included Shandong counties. Table S2. Hospital admissions, socioeconomic and health care accessibility indicators, and ambient NO2 concentrations of each area during the study period. Table S3. Meta-regression results for per capita GDP and hospital beds per thousand people of 39 counties in two-stage model analysis. Table S4. Attributable numbers and fractions of hospital admissions, total hospital stays and total expenses (thousand CNY) that can be reduced when the annual NO2 concentration reaches the WHO 2005 AQG. Fig. S1. Three-stage cluster sampling process, demographic characteristics of the sample population, and sample selection flowchart. Fig. S2. Concentration–response curve between NO2 concentrations (lag 0) and cardiovascular disease (a), coronary heart disease (b), ischemic stroke (c), and hypertension (d) hospital admissions. The vertical scale can be interpreted as the relative change in the mean effect of NO2 on mortality; the fraction of the curve below zero denotes a smaller estimate than the mean effect. Fig. S3. Comparison between the results of (a) subsample analysis in the aging population (aged above 60) and among (b) all study subjects. [file 12939_2024_2117_MOESM1_ESM.docx]

**Supplementary Materials**

**Urban‒rural disparities in the association of nitrogen dioxide exposure with cardiovascular disease risk in China:**

**Effect size and economic burden**

Yike Zhang ^1, 2†^, Mengxiao Hu ^1, 2†^, Bowen Xiang ^1, 2^, Haiyang Yu ^1, 2^, Qing Wang ^1, 2*^

^1^ Department of Biostatistics, School of Public Health, Shandong University, Jinan, 250012, Shandong, China

^2^ National Institute of Health Data Science of China, Shandong University, Jinan, China

^†^ Yike Zhang and Mengxiao Hu contributed equally to this work.

^*^ Correspondence: Qing Wang. E-mail: wangqing1984@126.com

Table S1. County names, county codes and sample sizes of the included Shandong counties.

Table S2. Hospital admissions, socioeconomic and health care accessibility indicators, and ambient NO_2_ concentrations of each area during the study period.

Table S3. Meta-regression results for per capita GDP and hospital beds per thousand people of 39 counties in two-stage model analysis.

Table S4. Attributable numbers and fractions of hospital admissions, total hospital stays and total expenses (thousand CNY) that can be reduced when the annual NO_2_ concentration reaches the WHO 2005 AQG.

Fig. S1. Three-stage cluster sampling process, demographic characteristics of the sample population, and sample selection flowchart.

Fig. S2. Concentration‒response curve between NO_2_ concentrations (lag 0) and cardiovascular disease (a), coronary heart disease (b), ischemic stroke (c), and hypertension (d) hospital admissions. The vertical scale can be interpreted as the relative change in the mean effect of NO_2_ on mortality; the fraction of the curve below zero denotes a smaller estimate than the mean effect.

Fig. S3. Comparison between the results of (a) subsample analysis in the aging population (aged above 60) and among (b) all study subjects.

Table S1.

County names, county codes and sample sizes of the included Shandong counties.

| City name | Area | County code | County name | Sample size |
| --- | --- | --- | --- | --- |
| Binzhou | urban | 371602 | Bincheng | 105,148 |
|  | rural | 371603 | Zhanhua | 223,519 |
| Dezhou | urban | 371402 | Decheng | 139,157 |
|  | rural | 371482 | Yucheng | 286,032 |
| Dongying | urban | 370502 | Dongying | 104,605 |
|  | rural | 370523 | Guangrao | 305,878 |
| Heze | urban | 371702 | Mudan | 109,529 |
|  | rural | 371721 | Caoxian | 185,609 |
| Jinan | urban | 370102 | Lixia | 159,852 |
|  |  | 370103 | Shizhong |  |
|  | rural | 370124 | Pingyin | 218,628 |
| Jining | urban | 370811 | Rencheng | 153,845 |
|  | rural | 370881 | Qufu | 301,034 |
|  |  | 370812 | Yanzhou |  |
| Laiwu | urban | 370117 | Gangcheng | 105,464 |
|  | rural | 370116 | Laicheng | 155,422 |
| Liaocheng | urban | 371502 | Dongchangfu | 98,489 |
|  | rural | 371521 | Yanggu | 2061,79 |
| Linyi | urban | 371302 | Lanshan | 127,673 |
|  |  | 370312 | Hedong |  |
|  | rural | 371323 | Yishui | 239,289 |
|  |  | 371325 | Feixian |  |
| Qingdao | urban | 370203 | Shibei | 363,140 |
|  | rural | 370215 | Jimo | 503,008 |
|  |  | 370211 | Huangdao |  |
| Rizhao | urban | 371102 | Donggang | 160,474 |
|  | rural | 371122 | Jvxian | 183,386 |
| Taian | urban | 370902 | Taishan | 111,423 |
|  | rural | 370923 | Ningyang | 170,715 |
| Weihai | urban | 371002 | Huancui | 216,108 |
|  | rural | 371082 | Rongcheng | 177,140 |
| Weifang | urban | 370705 | Kuiwen | 115,761 |
|  | rural | 370784 | Anqiu | 169,544 |
| Yantai | urban | 370611 | Fushan | 922,44 |
|  | rural | 370685 | Zhaoyuan | 290,736 |
|  |  | 370612 | Muping |  |
| Zaozhuang | urban | 370481 | Tengzhou | 69,063 |
|  | rural |  |  | 168,577 |
| Zibo | urban | 370303 | Zhangdian | 129,388 |
|  | rural | 370321 | Huangtai | 186,458 |

Table S2.

Hospital admissions, socioeconomic and health care accessibility indicators, and ambient NO_2_ concentrations of each area during the study period.

| City | County | Area | Hospital admissions | | | | Socioeconomic and health care accessibility indicators | | Average NO_2_ concentration  (μg/m^3^)  Mean (SD**)** |
| --- | --- | --- | --- | --- | --- | --- | --- | --- | --- |
|  |  |  | CVD | CHD | Ischemic Stroke | Hypertension | GDP per capita (CNY) | Number of beds per thousand people |  |
| Linyi | Feixian | rural | 3654 | 639 | 1790 | 383 | 35867 | 4.32 | 35.59 (13.23) |
|  | Yishui | rural | 6087 | 2730 | 1468 | 634 | 36857 | 6.61 | 35.73 (12.60) |
|  | Hedong | urban | 1853 | 490 | 461 | 399 | 35515 | 5.39 | 38.04 (15.55) |
|  | Lanshan | urban | 2177 | 466 | 684 | 396 | 65373 | 10.16 | 39.03 (15.16) |
| Weifang | Anqiu | rural | 2655 | 294 | 391 | 122 | 32807 | 4.71 | 36.10 (12.61) |
|  | Kuiwen | urban | 4662 | 1209 | 817 | 455 | 58156 | 23.41 | 36.10 (14.04) |
| Dezhou | Yucheng | rural | 9135 | 2358 | 3376 | 1161 | 50396 | 3.68 | 41.94 (15.75) |
|  | Decheng | urban | 8325 | 3552 | 2434 | 832 | 63446 | 4.50 | 40.41 (16.62) |
| Zaozhuang | Tengzhou | rural | 9201 | 672 | 2166 | 3367 | 64029 | 4.81 | 35.93 (12.51) |
|  |  | urban | 3921 | 435 | 860 | 1243 |  |  |  |
| Binzhou | Zhanhua | rural | 5750 | 900 | 2050 | 731 | 49979 | 3.98 | 36.90 (15.50) |
|  | Bincheng | urban | 6133 | 1102 | 2001 | 765 | 76309 | 13.21 | 39.17 (15.99) |
| Jinan | Pingyin | rural | 9762 | 2022 | 3301 | 1149 | 68866 | 5.17 | 41.14 (15.47) |
|  | Shizhong | urban | 7265 | 2653 | 1989 | 727 | 133558 | 8.47 | 42.84 (16.58) |
|  | Lixia | urban | 4041 | 1436 | 754 | 524 | 189768 | 30.60 | 46.38 (17.30) |
| Dongying | Guangrao | rural | 11067 | 2351 | 3582 | 621 | 153578 | 5.26 | 39.53 (15.24) |
|  | Dongying | urban | 2918 | 1016 | 341 | 253 | 153293 | 6.46 | 34.49 (15.37) |
| Jining | Qufu | rural | 7971 | 2656 | 2105 | 545 | 64839 | 4.20 | 39.38 (14.09) |
|  | Yanzhou | rural | 3903 | 1087 | 1003 | 320 | 107081 | 6.80 | 39.99 (14.44) |
|  | Rencheng | urban | 16212 | 7075 | 3085 | 1375 | 58808 | 4.63 | 40.24 (14.68) |
| Zibo | Huantai | rural | 6659 | 2466 | 1627 | 488 | 105146 | 7.67 | 46.25 (15.56) |
|  | Zhangdian | urban | 9653 | 4480 | 1670 | 1348 | 119207 | 11.80 | 51.24 (16.99) |
| Weihai | Rongcheng | rural | 5675 | 1327 | 706 | 679 | 166751 | 7.25 | 13.72 (5.71) |
|  | Huancui | urban | 8135 | 2426 | 600 | 2265 | 111228 | 4.22 | 22.31 (9.99) |
| Qingdao | Huangdao | rural | 11018 | 4192 | 1706 | 1788 | 183580 | 4.10 | 24.20 (9.54) |
|  | Jimo | rural | 4576 | 1235 | 1353 | 818 | 98606 | 4.35 | 25.59 (10.95) |
|  | Shibei | urban | 51396 | 14450 | 6812 | 12650 | 64201 | 11.26 | 21.83 (11.24) |
| Taian | Ningyang | rural | 4758 | 973 | 1655 | 427 | 53276 | 4.11 | 40.17 (14.30) |
|  | Taishan | urban | 7526 | 2757 | 2435 | 760 | 65040 | 13.92 | 39.35 (14.14) |
| Yantai | Zhaoyuan | rural | 7893 | 487 | 1651 | 868 | 121723 | 4.31 | 27.01 (11.45) |
|  | Muping | rural | 3733 | 180 | 666 | 435 | 70021 | 6.27 | 24.87 (10.54) |
|  | Fushan | urban | 2656 | 155 | 318 | 352 | 94271 | 6.14 | 29.37 (12.11) |
| Rizhao | Jvxian | rural | 3461 | 1090 | 852 | 345 | 34225 | 4.12 | 34.72 (12.55) |
|  | Donggang | urban | 5536 | 1930 | 811 | 822 | 61348 | 1.88 | 28.13 (10.58) |
| Heze | Caoxian | rural | 8964 | 3186 | 2250 | 819 | 19861 | 3.82 | 35.39 (13.25) |
|  | Mudan | urban | 8222 | 2699 | 2027 | 742 | 38568 | 2.17 | 37.96 (14.37) |
| Laiwu | Laicheng | rural | 6384 | 1515 | 2335 | 1293 | 55245 | 11.06 | 42.88 (14.80) |
|  | Gangcheng | urban | 5363 | 1531 | 1610 | 814 | 57535 | 21.10 | 41.67 (15.05) |
| Liaocheng | Yanggu | rural | 10259 | 2321 | 1243 | 1102 | 40018 | 4.02 | 40.89 (15.86) |
|  | Dongchangfu | urban | 4658 | 625 | 1093 | 332 | 58838 | 8.54 | 42.36 (17.10) |

Table S3.

Meta-regression results for per capita GDP and hospital beds per thousand people of 39 counties in two-stage model analysis.

|  |  | Estimates (95% CI) | P value | I² |
| --- | --- | --- | --- | --- |
| Model 0 | intercepts | - |  | 13.8% |
| Model 1 | + GDP per capita | 0.0001  (-0.0011, 0.0014) | 0.821 | 16.0% |
| Model 2 | + Hospital beds per thousand people | -0.0005  (-0.0014, 0.0003) | 0.215 | 12.8% |

Table S4.

Attributable numbers and fractions of hospital admissions, total hospital stays and total expenses (thousand CNY) that can be reduced when the annual NO_2_ concentration reaches the WHO 2005 AQG.

|  | **Attributable number (95% CI)** | |  | **Attributable fraction in % (95% CI)** | |
| --- | --- | --- | --- | --- | --- |
|  | **Urban** | **Rural** |  | **Urban** | **Rural** |
| **Cardiovascular disease** |  |  |  |  |  |
| Hospital admissions (case) | 327 (179, 475) | 185 (81, 289) |  | 0.46 (0.25, 0.66) | 0.26 (0.11, 0.40) |
| Total hospital stays (days) | 3689 (2012, 5354) | 1659 (725, 2590) |  | 0.47 (0.26, 0.69) | 0.26 (0.11, 0.41) |
| Total expenses (thousand CNY) | 4112 (2243, 5968) | 1249 (545, 1949) |  | 0.51 (0.28, 0.74) | 0.24 (0.10, 0.38) |
| **Coronary heart disease** |  |  |  |  |  |
| Hospital admissions (case) | 86 (-10, 180) | 87 (35, 138) |  | 0.33 (-0.04, 0.70) | 0.47 (0.19, 0.74) |
| Total hospital stays (days) | 843 (-95, 1771) | 702 (281, 1120) |  | 0.34 (-0.04, 0.72) | 0.48 (0.19, 0.76) |
| Total expenses (thousand CNY) | 1120 (-126, 2353) | 608 (243, 971) |  | 0.38 (-0.04, 0.79) | 0.42 (0.17, 0.67) |
| **Ischemic stroke** |  |  |  |  |  |
| Hospital admissions (case) | 85 (25, 145) | 49 (-4, 103) |  | 0.48 (0.14, 0.81) | 0.24 (-0.02, 0.50) |
| Total hospital stays (days) | 1076 (317, 1825) | 496 (-44, 1033) |  | 0.50 (0.15, 0.85) | 0.25 (-0.02, 0.51) |
| Total expenses (thousand CNY) | 916 (270, 1553) | 290 (-26, 603) |  | 0.53 (0.16, 0.89) | 0.23 (-0.02, 0.48) |
| **Hypertension** |  |  |  |  |  |
| Hospital admissions (case) | 69 (14, 122) | 41 (10, 72) |  | 0.87 (0.18, 1.54) | 0.55 (0.14, 0.97) |
| Total hospital stays (days) | 703 (144, 1250) | 327 (82, 570) |  | 0.89 (0.18, 1.58) | 0.56 (0.14, 0.97) |
| Total expenses (thousand CNY) | 522 (107, 928) | 134 (34, 234) |  | 0.93 (0.19, 1.66) | 0.53 (0.13, 0.92) |


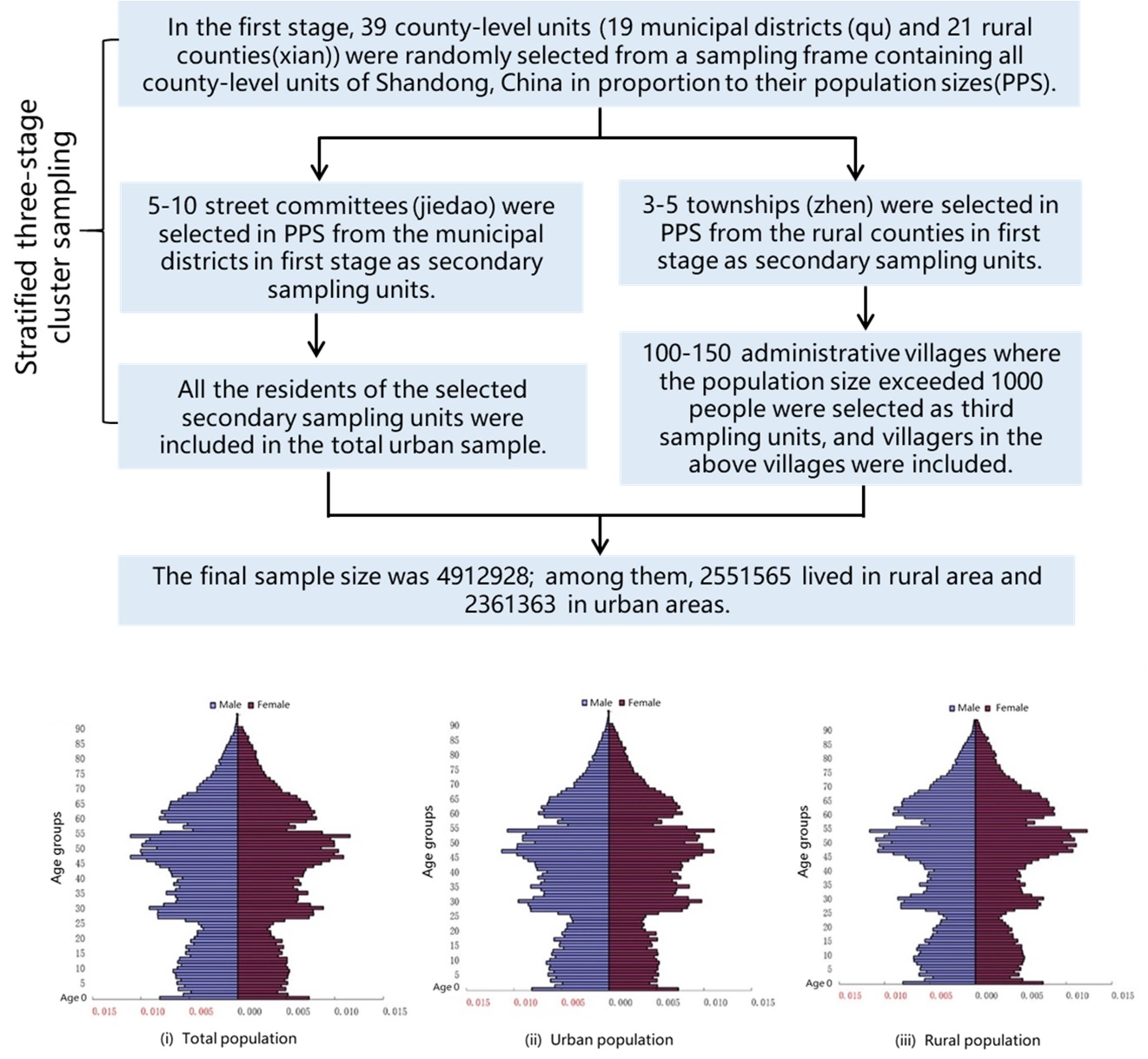


Fig. S1. Three-stage cluster sampling process and demographic characteristics of the sample population.


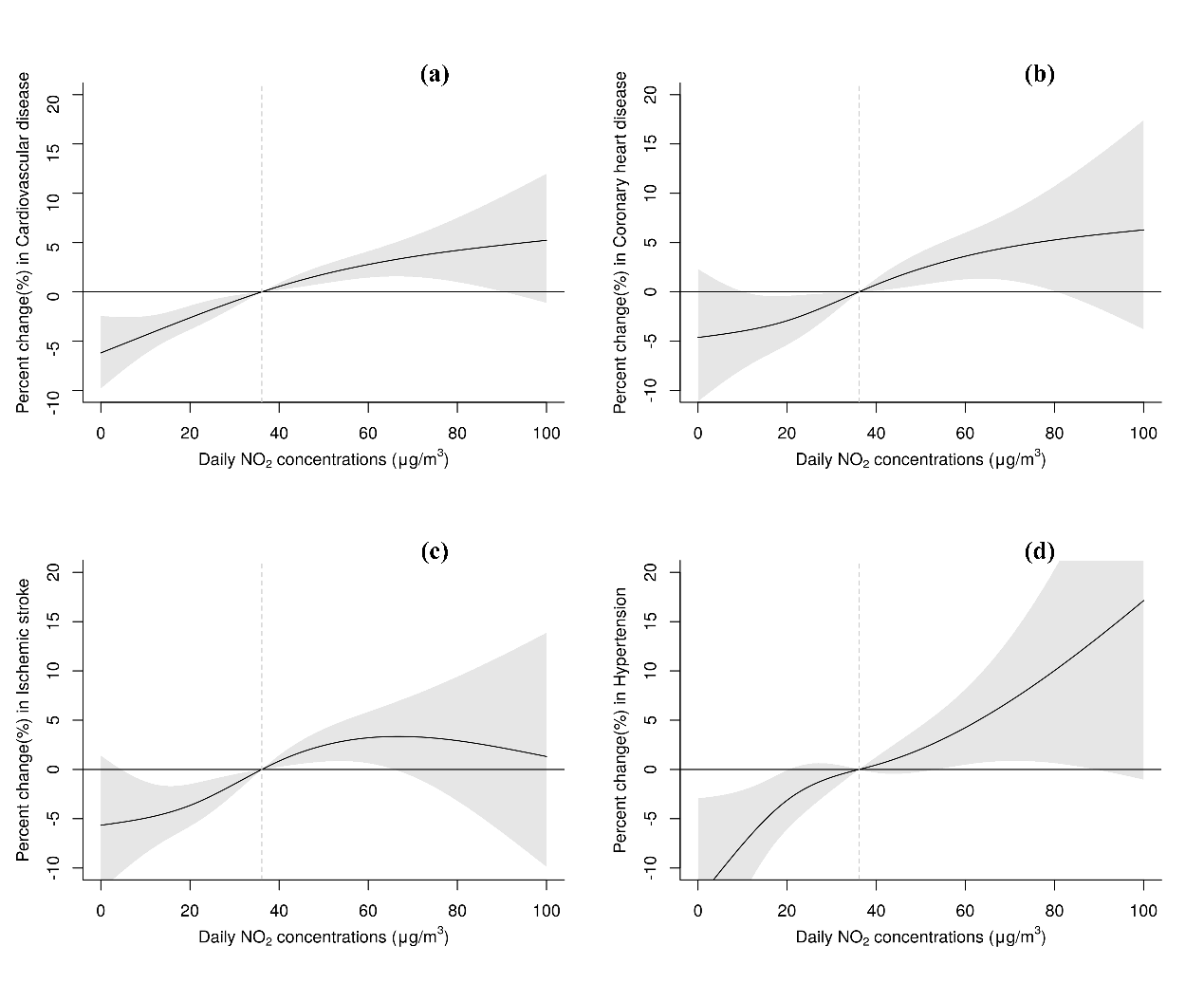


Fig. S2. Concentration‒response curve between NO_2_ concentrations (lag 0) and cardiovascular disease (a), coronary heart disease (b), ischemic stroke (c), and hypertension (d) hospital admissions. The vertical scale can be interpreted as the relative change in the mean effect of NO_2_ on mortality; the fraction of the curve below zero denotes a smaller estimate than the mean effect.


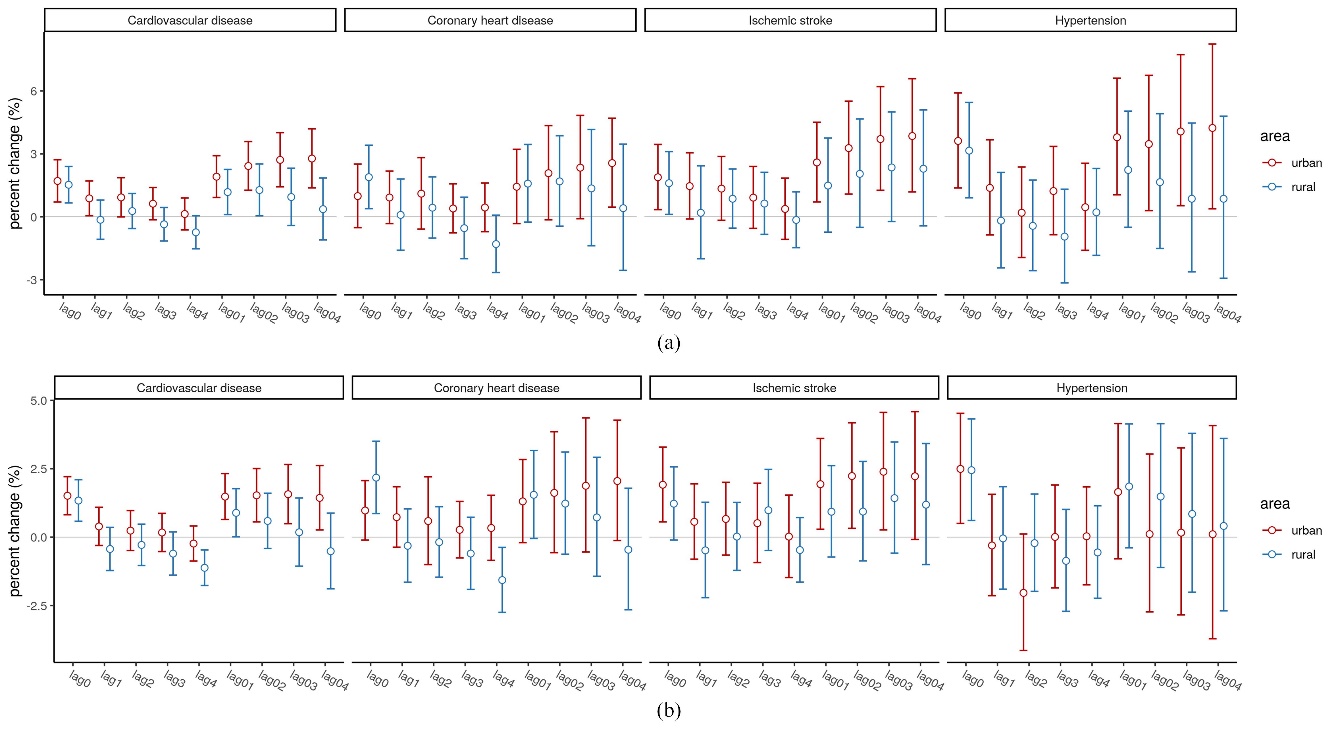


Fig. S3. Comparison between the results of (a) subsample analysis in the aging population (aged above 60) and among (b) all study subjects.
